# A Protocol for Enhancing Allied Health Care for Older People in Residential Care: The EAHOP Intervention

**DOI:** 10.3390/healthcare13030341

**Published:** 2025-02-06

**Authors:** Stephen Isbel, Nathan M. D’Cunha, Lara Wiseman, Paresh Dawda, Sam Kosari, Claire Pearce, Angela Fearon, Faran Sabeti, Jennifer Hewitt, Jane Kellett, Mark Naunton, Helen Southwood, Pip Logan, Ramanathan Subramanian, Neil H. Chadborn, Rachel Davey, Kasia Bail, John R. Goss, Ananthan Ambikairajah, Michelle Lincoln, Helen Holloway, Diane Gibson

**Affiliations:** 1 Centre for Ageing Research and Translation, Faculty of Health, University of Canberra, Bruce, ACT 2617, Australia; nathan.dcunha@canberra.edu.au (N.M.D.); lara.wiseman@canberra.edu.au (L.W.); paresh.dawda@nextpracticehealth.com (P.D.); angie.fearon@canberra.edu.au (A.F.); jane.kellett@canberra.edu.au (J.K.); helen.southwood@canberra.edu.au (H.S.); kasia.bail@canberra.edu.au (K.B.); ananthan.ambikairajah@canberra.edu.au (A.A.); helen.holloway@canberra.edu.au (H.H.); diane.gibson@canberra.edu.au (D.G.); 2 School of Rehabilitation and Exercise Sciences, Faculty of Health, University of Canberra, Bruce, ACT 2617, Australia; claire.pearce@canberra.edu.au; 3 Next Practice Deakin, Canberra, ACT 2600, Australia; 4 School of Rehabilitation Health Sciences, Faculty of Health, University of Canberra, Bruce, ACT 2617, Australia; sam.kosari@canberra.edu.au (S.K.); faran.sabeti@canberra.edu.au (F.S.); mark.naunton@canberra.edu.au (M.N.); 5 University Centre for Rural Health, Lismore & University of Sydney, Sydney, NSW 2006, Australia; jennifer.hewitt@sydney.edu.au; 6 School of Medicine, University of Nottingham, Nottingham NG7 2UH, UK; pip.logan@nottingham.ac.uk (P.L.); neil.chadborn@nottingham.ac.uk (N.H.C.); 7 Faculty of Science and Technology, University of Canberra, Bruce, ACT 2617, Australia; ram.subramanian@canberra.edu.au; 8 Health Research Institute, University of Canberra, Bruce, ACT 2617, Australia; rachel.davey@canberra.edu.au (R.D.); john.goss@canberra.edu.au (J.R.G.); 9 School of Nursing, University of Canberra, Bruce, ACT 2617, Australia; 10 Synergy Nursing and Midwifery Research Centre, ACT Health, Canberra, ACT 2605, Australia; 11 National Centre for Epidemiology and Population Health, Australian National University, Canberra, ACT 2601, Australia; 12 Discipline of Psychology, Faculty of Health, University of Canberra, Bruce, ACT 2617, Australia; 13 School of Psychology, The University of Sydney, Sydney, NSW 2050, Australia; 14 Brain and Mind Centre, The University of Sydney, Sydney, NSW 2050, Australia; 15 University of Canberra, Bruce, ACT 2617, Australia; michelle.lincoln@canberra.edu.au

**Keywords:** allied health, nursing home, care home, residential aged care, health services, multidisciplinary, allied health assistants, falls, frailty, quality of life

## Abstract

Background: Complications due to frailty and injury after falls are a significant problem for people living in residential aged care. This can lead to a range of negative outcomes including poor physical, social, and psychological well-being. The Australian Royal Commission into Aged Care Quality and Safety highlighted many aspects of care in residential aged care homes requiring improvement, leading to specific recommendations aimed at improving the outcomes in this area. This contributed to four recommendations calling for increased allied health interventions to meet the unmet needs in residential aged care. This intervention aims to implement and evaluate evidence-based allied health interventions for people living in residential aged care specifically relating to frailty, preventing falls, and maintaining engagement in everyday activities. Method: This is a pragmatic, non-randomised, pre–post design study where six groups of up to 10 residents of an aged care home will start the intervention at staggered times. The EAHOP intervention is an integrated application of a suite of allied health services (occupational therapy, physiotherapy, dietetics, speech pathology, pharmacy, and optometry), with general practitioner involvement, using allied health assistants in an integrated transdisciplinary model of care. The baseline period is 6 weeks, and intervention is a maximum of 36 weeks with follow-up at 12 and 24 weeks. Primary outcomes measure changes in falls, frailty, and quality of life. A qualitative program evaluation will be completed as well as an economic analysis. Conclusion: The results of the study will provide information about the clinical, implementation, and effectiveness outcomes of this integrated, transdisciplinary allied health service model for people living in residential aged care. The results will be used to develop evidence-informed guidelines for residential aged care providers on the delivery of allied health services.

## 1. Background

In Australia in 2023, 185,127 people were living in residential aged care [1] and complications due to frailty and injury after falls are a significant problem for these residents. Following entry into residential care, 3.7% of people are hospitalised due to falls within 90 days, and 9.8% within a year [2]. Every six months, it is estimated that 50% of people with dementia in residential aged care experience a fall [3], leading to significant financial and social costs [4]. Additionally, around 95% of residents are estimated as being frail or pre-frail [5], which is also associated with negative health outcomes resulting in hospitalisation [6]. People living in residential aged care also often find a discontinuity between what they found meaningful and engaging prior to entering aged care and what they experience in care, which adversely affects their quality of life [7]. There is a need to focus on enabling people in residential aged care to keep engaging in activities they find meaningful [8] and maximising engagement in everyday life.

The Royal Commission into Aged Care Quality and Safety heard that only 24% of people living in residential aged care felt their needs were always met across several care experiences [9]. This contributed to four recommendations calling for increased allied health interventions to meet the unmet needs in residential aged care. Recommendation 37 specifically lists allied health as a service that should be provided in residential care. Recommendation 38 lists thirteen allied health professionals providers should employ or retain. Recommendation 61 recommends changes to the Medicare Benefits Schedule to include allied health service provision in residential care. Recommendation 69 states that allied health care should generally be provided by aged care providers.

Allied health has an essential role in meeting the challenges associated with fall prevention, addressing frailty and keeping residents engaged in meaningful activities [8,10]. The Royal Commission received submissions comparing residential aged care in British Columbia where residents receive 22 min of allied health service per day, to Australia where only 2% residents receive this level of service [11]. Even after significant reform following the Royal Commission, there has not been any improvement in the amount of allied health services in residential aged care, and in fact this appears to have further deteriorated [12]. There is a clear and urgent need for a new model of allied health provision in residential aged care.

This intervention aims to implement and evaluate evidence-based allied health interventions for people living in residential aged care specifically related to frailty, preventing falls, and maintaining engagement in everyday activities. The intervention, conducted in partnership with General Practitioners (GPs) and a residential aged care provider, will coordinate an integrated transdisciplinary program (EAHOP intervention) of evidence-based allied health services including occupational therapy, physiotherapy, speech pathology, pharmacy, dietetics, and optometry to older people living in residential care, in conjunction with the nurses and care workers onsite. Allied health assistants will also be involved in delivering transdisciplinary interventions, under the supervision of the relevant allied health professional.

### 1.1. Research Questions

1. Is an integrated transdisciplinary allied health service model effective in preventing falls, addressing frailty, and promoting engagement in people transitioning to or living in residential aged care?
2. Can an integrated transdisciplinary allied health service model improve outcomes focused on falls, frailty, and engagement, compared to baseline measures, in people transitioning to or living in residential aged care?
3. What are the care experiences of residents, carers, staff, and other key stakeholders when an integrated transdisciplinary allied health service model is implemented in residential aged care?

### 1.2. Objectives

1. Implement and evaluate a new integrated and transdisciplinary model of allied health intervention in relation to the impact on falls, frailty, engagement, quality of life, and cost-effectiveness.
2. Evaluate the process (or implementation) of the new allied health service model by asking key stakeholders (residents, families, care staff, and nursing staff) about their experiences of participating in the model.
3. Produce evidence-informed guidelines for residential aged care providers in Australia to implement an integrated transdisciplinary allied health service.

## 2. Methods

### 2.1. Study Design

This is a pragmatic, non-randomised, pre–post design study where six groups of up to 10 participants will start the intervention at different times. Each group will commence a baseline period lasting six weeks. Primary outcome measures will be administered in week 0 (−t_1_) of the baseline period and in week 6 (−t_2_) of the baseline period. The allied health professionals will conduct assessments using the secondary outcome measures during the baseline period, making recommendations for consideration at the case conference held with the resident’s usual General Practitioner. The prescribed allied health intervention/s will last a maximum of 36 weeks with primary outcome measures taken during the intervention period at 12 (t_1_), 24 (t_2_), and 36 (t_3_) weeks, then at 12 (t_4_) and 24 (t_5_) weeks post-intervention. The case conferences will take place in week 1 or week 2 of the intervention period. The timing of the secondary outcome measures will vary depending on the mix of interventions chosen by the allied health professional and the participant needs. See the Supplementary file for further details.

### 2.2. Study Setting

The intervention will be conducted in a residential aged care home in Canberra, Australia, with a maximum of 60 residents.

### 2.3. Participants

Any new or existing (permanent) resident of the residential aged care home is eligible. Staff at the home will approach any new or existing resident and/or family with a study flyer. The research team will present the study to the residents at the regular residents’ meeting. Participants will register their interest through a register kept by the nursing staff at the home and/or they will be able to contact the research team through contact details listed on the flyer and promotional material posted at the home. All participants and/or their carers, power of attorney, or guardian will be required to give written consent after reading the relevant participant information at which time the Clinical Liaison Manager (who is a Registered Nurse) will consent them into the study and assign them a study number. Residents with a diagnosis of dementia or identified by care staff as experiencing cognitive impairment are eligible. All participants will be screened for cognitive function using the Psychogeriatric Assessment Scale (PAS) [13]. There is no cutoff score for inclusion; however, in some cases, people with severe cognitive impairment will not be able to engage in the allied health program. For example, people who have severe expressive and receptive communication impairment and who are immobile due to dementia. For those residents with severe dementia or on an end-of-life pathway, we will liaise with the relevant medical team and/or staff Registered Nurse to determine if these residents should ethically be considered for the program. Residents who elect not to participate in the intervention will receive their usual medical, health, and lifestyle services as usual.

Participants will be able to discontinue the intervention at any time at their, or their Enduring Power of Attorney’s, request. Participants will be withdrawn from the study if any deterioration in their medical condition makes participation unsafe in the opinion of their General Practitioner.

### 2.4. Conceptual and Operational Framework

#### 2.4.1. Model of Care

The EAHOP intervention has been designed following the Institute for Healthcare Improvement [14] 4Ms framework of an age-friendly health system as the model of care to organise allied health interventions. The 4Ms are as follows:

- What Matters: Know and align care with each older adult’s goals and care preferences.
- Mentation: Prevent, identify, treat and manage dementia, depression, and delirium across care settings.
- Medication: If medication is necessary, it should not interfere with What Matters to the older person.
- Mobility: Ensure that older people move safely every day.

#### 2.4.2. Case Conferencing

The allied health professionals will conduct their discipline-specific, secondary outcome measures during the baseline period and then make recommendations to be discussed at a case conference. The case conference will be attended by the resident’s General Practitioner, the research clinical liaison manager, and where possible the resident and family member. A plan of action will be agreed with the resident and their family following the case conference that could involve one or more allied health interventions depending on the needs of the resident.

#### 2.4.3. Clinical Liaison Manager

A Clinical Liaison Manager (Registered Nurse) will be embedded within the aged care home with the overall goal of ensuring the case conferences and interventions are implemented as intended, managing data collection and providing a central point of contact for communication between the participants and their family members, aged care team, the embedded allied health team, and the research team.

#### 2.4.4. Allied Health Professionals and General Practitioner

There are six allied health professionals, a dietitian, pharmacist, physiotherapist, occupational therapist, optometrist, and speech pathologist, who will conduct discipline-specific assessments and provide discipline-specific interventions as needed, as identified and agreed in the case conference with a General Practitioner assisting with the coordination of care.

#### 2.4.5. Allied Health Assistants

Allied health assistants will work directly with five of the allied health professionals (dietitian, occupational therapist, physiotherapist, dietitian, and speech pathologist) to implement the prescribed allied health interventions.

**Figure 1:**
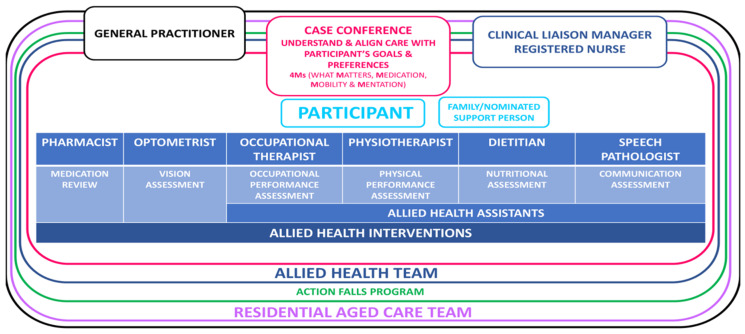
An integrated transdisciplinary allied health service model (EAHOP intervention).

See Figure 1 for a diagrammatical representation of the EAHOP intervention. 

### 2.5. Intervention

A brief description of the discipline-specific interventions is described in Table 1.

#### Action Falls Program

In addition to the allied health interventions for individual participants (described in Table 1), the intervention will also involve an organisational intervention through implementation of the Action Falls program [17]. An Action Falls checklist will be completed with each participant (and or carer/family member, as appropriate). The Clinical Liaison Manager, who will receive formal training in the implementation of the Action Falls Program, will oversee the completion of the checklist, which can be carried out by any member of the team or residential aged care staff once training is complete.

### 2.6. SampleSsize

The sample size was calculated using R based on 1000 simulated versions of the study, where the power estimate is the proportion of simulated studies that gave *p* < α for the difference between the pre- and post-intervention stages. Using the Short Physical Performance Battery (SPPB) as the primary outcome measure with 50 subjects, the increased mean responses anticipated with alpha 0.01 are shown in Table 2. This is assuming the following parameters: average response at baseline 5; between-subject SD 2.5; variability in effect between subjects SD 2 (so the effect is 2.5 with SD of 2); within-subject SD of 1; and between-group SD of 2 [18].

We expect up to 60 existing residents will consent to the intervention. The site estimates that 20 new residents will enter the home during the study period, and we estimate 15 of these new residents will consent to the study. With a 20% dropout and loss to follow-up, the highest number of participants to complete the intervention is 60 participants.

### 2.7. Data Collection and Monitoring

Data collection forms will be available on the RedCap data management system where the relevant allied health professional or Clinical Liaison Manager will enter the data as each outcome measure is completed. Data from participants who do not complete the intervention will be collected up until the time they withdraw from the study. An independent Project Governance Committee will be established to meet quarterly with a function to monitor the data collection procedures and to monitor reported adverse events. Adverse events will be reported by any member of the research team or the aged care provider on an adverse events form that will be stored electronically on a secure server and available in hard copy from the aged care provider.

### 2.8. Outcome Measures

Primary outcome measures are the number of residents who fall (falls) and the number of falls for each participant (fall rate) in 12 months prior to enrollment in the project. All falls are recorded by the residential aged care home and these data will be accessed by the research team. Other outcome measures are the Quality of Life Aged Care Consumers tool (QOL-ACC) [19], The Frail in Nursing Homes (Frail-NH) Scale [20], and the Short-Form Physical Performance Battery (SPPB) [21]. Each allied health discipline has discipline specific outcome measures that are secondary outcome measures along with a measure of pain. See Table 3.

### 2.9. Program Evaluation

An implementation/program evaluation of this new transdisciplinary allied health service model (EAHOP intervention) to address poor outcomes associated with falls and frailty for people entering and living in residential aged care will also be conducted. See Table 4 for details. The evaluation objectives are as follows:

1. To ascertain if the transdisciplinary model has met the project aim of improving quality of care.
2. To identify barriers and enablers to implementation of the program as planned, with the aim of developing an implementation guideline.
3. To provide a detailed description of program resourcing.

**Table 4:** Summary of the evaluation process.

Implementation Outcome	Measurement Details
Feasibility	Descriptive: size of RAC home and staffing Description of all interventions delivered Cost per client (of total allied health service and per allied health service) Qualitative data from key stakeholder interviews
Access and Uptake	Number of referrals Number of consented participants Occasions of service (how many times seen) per allied health discipline (AH) and allied health assistant (AHA) per resident and discipline Units of service, direct and indirect time in minutes per resident and discipline Time spent on case conferences
Acceptability	Interviews re: experiences of stakeholders Service managers Care home staff ×10 Allied health professionals (all) Allied health assistants (all) Carers ×15 Participants ×15 GP/s

### 2.10. Economic Evaluation

An economic evaluation of the EAHOP intervention will be measured by calculating the Quality-Adjusted Life Year (QALY) using data from the QOL-ACC. The costs of staff (including on-costs and overhead costs) involved in the intervention will be enumerated, and cost savings due to expected reductions in hospitalisations and falls will be estimated by comparing the RAC hospital leave days for those involved in the intervention as compared to an age-, sex-, and level-matched sample.

### 2.11. Fidelity Assessment

Fidelity to the intervention will be assessed using Hasson’s Conceptual Framework [33]. The percentage of consented participants triaged through case conferencing will be reported. At the discipline level, at least 10% of all participants will be randomly sampled and evaluated for accuracy against content, frequency, and duration of intervention. Fidelity rating at the intervention level and discipline level will be reported as high/medium/low.

## 3. Data Analysis

### 3.1. Quantitative Analysis

All statistical analysis will be conducted using R version 4.2.1 (23 June 2022) in Rstudio (version 2023.6.2.561). Baseline demographic and descriptive statistics will be summarised for each group prior to the intervention starting (control phase). Primary outcome measures will be collected twice in the control period at week 0 and week 6. Primary outcome measures will be collected every 12 weeks during the intervention (weeks 12, 24, and 36) and at 12 weeks and 24 weeks post-intervention for each group. Categorical data will be summarised using frequency statistics and will be visualised. Continuous and other numerical data will be visually explored and summarised using sample descriptive statistics, selected as appropriate based on apparent distributions. A mixed-effects model will be computed, with treatment stage (i.e., pre- vs. post-intervention) as a fixed effect and subject as a random effect. Sensitivity analyses will be conducted, which will impute missing data using the “mice” package (version 3.15.0) [34].

### 3.2. Qualitative Analysis

We will purposefully sample participants, carers, aged care staff, and health professionals as described in the program evaluation. In-depth interviews will be conducted to elicit their thoughts, opinions, and experience of the intervention. All interviews will be transcribed verbatim, and a reflexive thematic analysis will be completed using a variation of Braun and Clark’s reflexive thematic analysis [35]. Field notes will be kept and reported from key members of the research team for the duration of the study recording key events, interactions, and justification of key decisions made.

## 4. Ethics Review

This study has been approved by [blinded for peer review]. Any unforeseen amendments to the study protocol will only be enacted after expressed written approval from the HREC. Any important changes to the protocol will result in an ethics amendment to be approved with notifications to the ANZCTR and key stakeholders (participants, families, and the aged care provider).

## 5. Discussion

The results of this study will provide information about the clinical, implementation, and effectiveness outcomes of this integrated transdisciplinary allied health services model (EAHOP intervention) for people living in residential aged care and those transitioning into residential aged care. The results will be used to develop evidence-informed guidelines for residential aged care providers on the delivery of allied health services. The economic evaluation will provide evidence to inform how allied health service provision could be provided in residential aged care.

## Figures and Tables

**Table 1:** Summary of interventions within EAHOP.

	Description
Occupational Therapy	Participants will identify goals relating to activities of everyday living with specific reference to self-care, leisure activities, and productivity that are meaningful to them that they would like to be engaged with and/or become more satisfied with. The occupational therapist will develop goal-directed and individually tailored occupational therapy interventions to improve performance and satisfaction with the identified occupational performance challenges. These will be discussed with Health and Lifestyle staff to support ongoing sustainable implementation with the individual.
Physiotherapy	Participants will engage in exercise based upon the SUNBEAM program [15] involving 2 h per week of individually tailored strength and balance exercises prescribed by the physiotherapist for 25 weeks (total 50 h) and then a maintenance program for the duration of the intervention. In some instances, participants may not be suitable for the SUNBEAM program. In these cases, they will be offered an individualised virtual cycling experience [16] once a week for the intervention’s duration.
Dietetics and Nutrition	Participants diagnosed with weight loss or at risk of malnutrition will be provided with an individualised, high-protein, high-energy diet by fortifying menu items such as porridge, soups, mashed potato, and desserts, and/or oral nutrition supplements. In addition, the dietitian will provide modified menus to Food Service staff and training to Food Service staff on how to fortify menu items as per modified menus.
Speech Pathology	Participants will receive individualised interventions focused on communication partner training over a period of up to 36 weeks. The speech pathology intervention may include communication repair strategies, an education component, role play, practice, and recording of communication interactions to identify and address communication challenges.
Optometry	Participants will receive an onsite, mobile assessment by an optometrist evaluating visual function (including refraction, confrontation fields, visual acuity, ocular structure (macula and optic nerve lenticular assessment, and intra-ocular pressure) to address visual impairments that may contribute to fall risk. Actions arising from the assessment of visual function may include new spectacle prescriptions or referral of participants for management of ocular disease.
Pharmacy	Participants will receive an onsite medication review by a pharmacist who will provide recommendations regarding the resident’s medication to the participant’s GP, nursing staff, and family members. After the initial medication review, participants will receive a 2-week follow-up visit to understand how medication decisions are implemented in the medication treatment and charts. Each participant will have an initial case conference and then a follow-up visit from the pharmacist every 3 months (online or in person).

**Table 2:** Associated power based upon *n* = 50 and mean change in the SPPB.

Increase Mean Response After Intervention	Power
2.5	0.99
2	0.94
1.75	0.84
1	0.63

**Table 3:** Outcome measure summary.

Primary Outcome Measures	
Falls: Number of falls in past 12 months Number of falls during intervention period Number of falls after intervention	
Quality of Life Aged Care Consumers (QOL-ACC) [19]	
Frailty: The Frail in Nursing Homes (Frail-NH) Scale [20] and the and Patient Health Questionnaire (PHQ9) [22]	
Short Physical Performance Battery (SPPB) [21]	
**Secondary outcome measures**	**Allied health discipline**
Short Falls Efficacy Scale—International (Short FES-I) [23]	Physiotherapy
Subjective Global Assessment (SGA) [24] Mid-arm muscle circumference [25,26] Quantitative analysis of dietary intake will be calculated before and after fortification	Nutrition and Dietetics
Canadian Occupational Performance Measure (COPM) [27]	Occupational Therapy
Number of medications (that contributed to increased risk of falls) ceased/changed at case conferences Number of case conferences and pharmacist follow-ups Rate of GP agreement on pharmacist recommendations	Pharmacy
Number of new or updated spectacles recommended Number of referrals arising from vision assessment The National Eye Institute Visual Functioning Questionnaire—25 (VFQ-25 + 10) [28]	Optometry
Holden Communication Scale [29] Number of communication breakdowns occurring The number of resolutions (strategies used to repair the communication breakdowns)	Speech Pathology
Revised Index for Social Engagement (RISE) [30]	All
Brief Pain Inventory (short form) [31] or PAINAD [32]	All
ACTION Falls: number of ACTION falls checklists completed [17]	All
Hospital leave days	All
Program satisfaction survey	All
Qualitative interviews	All
Program evaluation	All
Economic analysis	All

## Data Availability

The datasets used and/or analysed during the current study are available from the corresponding author on reasonable request pending approval from the stated human research ethics committees.

## Supplementary Materials

The following supporting information can be downloaded at: https://www.mdpi.com/article/10.3390/healthcare13030341/s1, Scheme S1: EAHOP schedule of enrolment, interventions, and assessments.
