# Supplementary material for: A Protocol for Enhancing Allied Health Care for Older People in Residential Care: The EAHOP Intervention
Source: Healthcare (Basel). 2025 Feb 6;13(3):341. doi: 10.3390/healthcare13030341 (PMC11817486; doi:10.3390/healthcare13030341)
Supplement: Supplementary file 1 [file healthcare-13-00341-s001.zip › healthcare-3386343-supplementary.pdf]

Supplementary file:

A protocol for Enhancing Allied Health for Older People in Residential Care: the EAHOP (RC) intervention.

Scheme S1. EAHOP schedule of enrolment, interventions, and assessments.

	STUDY PERIOD						
	**Baseline**		**Intervention**			**Follow-Up**	
**TIMEPOINT**	$-t_1$	$-t_2$	t_1	t_2	t_3	t_4	t_5
**ENROLMENT:**							
**Eligibility screen**	X						
**Informed consent**	X						
**Case conference**			X				
**Allocation**	X						
**INTERVENTION:**							
Multidisciplinary Intervention				←			
**ASSESSMENTS:**							
Primary outcome measures	X	X	X	X	X	X	X
Secondary Outcome measures	X	X		←			
